# Treatment of Mammary Abscess

**Published:** 1881-04

**Authors:** Hiram Corson

**Affiliations:** Conshohocken, Pa.


THE
### Independent Practitioner.

Vol. II.—*April, 1881.*—No. 4.

ARTICLE I.

### THE TREATMENT OF MAMMARY ABSCESS.

BY HIRAM CORSON, M. D., Conshohocken, Pa.

Mr. Editor: There are few diseases which, while not being dangerous to life, give more suffering to the nursing woman than inflammation of the breast, which results in suppuration. The fact that it is not generally fatal is, perhaps, one reason that physicians give slight heed to the appeals of the patient for relief, and often treat it with remedies well known to be very inefficient, or abandon it to time, patience and the nurse. Indeed, physicians are sometimes charged with not desiring to hasten recovery, in as much as it is an opportunity to "run up a bill," without incurring great responsibility. In view of the great suffering borne by women under this affliction, at the very time when she most needs rest and comfort, and the inadequacy of the means, in common use, to allay inflammation of the breast, induced me to write a paper on the subject, to inform the members of the Philadelphia Obstetrical Society of my experience with the use of ice, or iced water, during many years of practice. The paper was read by Dr. Wm. Goodell; was favorably received by the Society and published in its "Transactions." As articles thus disposed of do not meet the eyes of many general practitioners, and as I believe, that if the means recommended were known to physicians generally, they would save many women from great pain and long suffering, it has occurred to me to offer it to you for publication in the Independent Practitioner.

In the Transactions of the Obstetrical Society of Philadelphia, for the year 1879, I found an article on Mammary Abscess; in the discussion ot which subject many members of the Society took part. During this discussion various means were recommended to prevent the formation of pus in the breast; to prevent and cure sore nipples, and to treat the breast after suppuration had taken place. Seven eminent physicians participated in this discussion, and as they are all persons much engaged in obstetrical practice, we may conclude that we have in this paper a brief account of the most approved modes of practice. I say modes, for no two of them approved of exactly the same plan or mode of treatment.

One of the speakers said that "the old fashion gathered breast is very seldom seen at the present time;" but he referred to "small tumors arising near the periphery of the gland; the lump is quite tender, treatment is applied and it is relieved, but returns and *in spite of all treatment* will finally break." "It occurs," he says, "in women who are nursing and who are doing well, and is, sometimes, the effect of exposure to sudden chill." The perfection to which nursing of the lying-in women has been brought, in our great cities, is probably one great cause of the scarcity of mammary abscess there. As we of the country have indifferent, unskilled nurses, and as many of the patients suffer from adverse surroundings, and, too, as we do not make many visits after the child is born, the disease frequently presents itself, and has often made great advances before it is brought to our notice; and yet, I ought to say, that it is not so frequent now as thirty or even twenty years ago, owing, perhaps to our having better nurses and giving more attention to the patients ourselves. In the long run of fifty years it was my fortune to have many cases of "inflammation of the breast" to treat, and great suffering have I witnessed while I was anxiously and faithfully applying the means now recommended by those who took part in the discussion to which I have referred. The plans of treatment seem to have undergone no change, the principles of treatment are the same, differing only in the strength of various ointments, or washes, or in the substitution of one astringent or poultice for another, as suited the different practitioners. The use of the fluid extract of phytolacca, as recommended by Dr. J. G. Allen, to remove congestion of the breast, is to me a new remedy; one I have never tried, and, as he does not claim for it any value save in the congestive cases, indeed, says, in none of the other forms which he notices "will it be found of the slightest use whatever," we may regard it as a means of cure of very limited advantage. Many of the speakers spoke as men who had been sorely tried in treating the sore nipples and inflamed mammary gland, and my sympathies were with them, for many a time I had experienced similar disappointments in the use of my remedies. Many years ago, in 1827–8, Prof. Wm. P. Dewees strongly urged upon us the use of flannels steeped in hot vinegar and re-applied frequently, as a means of removing the congestion, spoken of by Dr. Allen, and for the dispersion of which he recommends the phytolacca. In our first cases we resorted to it; then came the camphor melted in lard and applied by every nurse as a specific in inflamed breast, "to prevent it from gathering." Solutions of sugar of lead, lead plasters, and poultices followed the failure of the former remedies. In some cases we were not called until the abscess had opened, but in most of them the lancet was used. The breast was opened, the matter discharged, and sometimes a speedy cure followed; in other cases there was prolonged suffering, one abscess following another, until almost every part of the gland was unfit for use. In 1853, I was in attendance on a young, healthy woman with her third child. Two months prior to her confinement, she had an abscess of the breast, like those spoken of in the discussion by Dr. Albert Smith, which I had lanced and from which she had entirely recovered. A few days after the birth of her child I was called to see her, and found her suffering greatly from an inflammation of the left mamma—one of Dr. Allen's congestive cases. It was large, tense, and fiery red, over the most affected part. I at once proposed to her to cover it with cloths wet with iced water, and renewed every few minutes. She expressed a fear that it would give her a chill, but finally consented; they were applied, and the great comfort, produced in a short time, encouraged her to keep the nurse steady to the work. Finding it so grateful, and the relief so decided, I procured a bladder, and, putting some cold water and ice in it, had it put on the inflamed part. A few hours showed us that the danger of suppuration had passed. From that time until the present, twenty-seven years, I have used no other remedy, and, whenever called before suppuration has taken place, can always prevent it. So greatly was this patient relieved that she spoke of the remedy in such terms of praise as to make it a desirable remedy to any others in the town who feared a "gathering." To show that it is just as applicable, efficient, and safe in cases that have suppurated and been poulticed, and have "broke," or been lanced, one abscess being followed by another and another, until nearly the whole breast was involved, I will give an account of a case which occured in my practice.

Mrs. B——, a few days after her confinement, suffered from chill followed by pain, heat, redness, and swelling in the right breast; the nurse worried with it in the usual way, but the great suffering of the patient induced them to send for me. I had gone away for a week, and a medical friend took charge of her for me. He found her suffering greatly with a large abscess ready to be opened. It discharged freely and the poultice, which she had on at the time, was replaced. He saw her several times before my return, and opened another abscess, and still continued the poultice. My first visit to her was with the physician. She was suffering greatly. The breast was much swelled, was solid and heavy in some parts, and a red, highly inflamed surface of several inches, on the under and outer part, gave warning of a third abscess. I advised the use of ice, which greatly surprised both patient and physician. The fact that she had been kept very warm for two weeks, for fear of getting more chills, and that she had had warm poultices steadily applied during nearly all that time, was, to their minds, strong reason for objecting to its application—the great change from heat to cold they deemed most hazardous. As the patient was a new-comer in the place and knew nothing of the cold treatment, and positively refused to have it applied, the breast was supported and the poultice continued. She was truly wretched; half sitting up, supporting her suffering side, no good sleep, no appetite, the breast stinging and burning night and day, as those only know who have suffered like torment, she was a picture of distress. I concluded to bide my time, knowing, full well, that she could not hold out long against her sufferings and my persuasions. In a few days I opened the third abscess; the other openings, two, were still discharging and had become larger. I then prevailed on her, after the most solemn assurance that no harm would come to her, to have the ice applied. A large bladder was partly filled with water and lumps of ice and applied; two thicknesses of wet muslin first being applied to the inflamed breast. The relief was soon apparent to her, by the speedy removal of the great heat, which night and day had tortured her. That night she had comfort. There were no more abscesses; the heat, tension, and pain of the inflamed parts subsided, and in a few days, the hot, tender, angry breast was so changed that she rapidly regained her cheerfulness and health. Since that time she has been most earnest in praise of cold treatment—not one moment of comfort did she have before it was applied—and then what comfort! I saw her only a few months since, when she referred to it, she said: "Doctor, no tongue can tell how I suffered before the cold water and ice were applied, I believe they saved my life."

During the twenty-seven years that I have used it, I have very often been called to women whom I have found with a breast painful, swelled, and red over the swelled part; the result, the patient would tell me, of "a chill, which had happened in the night and fell right away on the breast," since which time she had had no rest. I here at once applied the ice, and in no instance, if suppuration had not already taken place, have I failed to disperse the inflammation, at the same time that I brought comfort to the patient. In some cases I have found the suppurating process so far advanced that nothing could prevent it, but even here I apply the ice, knowing that it will give the woman great comfort, by removing the heat, allaying the inflammation and thus preventing any more of the breast from becoming involved in the suppurating process. And now, as I write this sentence, I feel that some readers will stop here and read it over again. Then they will say: "*That* is ridiculous; the abscess has its bounds marked out early; treat it as you may, it will have a fixed size; no treatment will make it larger or smaller."

Several years ago, I was called to a case, and found a breast ready for the lancet, indeed, it was "dead ripe." It was evident from the blue color of the skin that it would soon slough if not opened, and indeed, even if it were; but no persuasion of mine to have it opened could prevail with her, because she had heard, "that if left to open itself it would not gather again." So I was forced to leave her, warning her though that she would be likely to lose her whole breast. In a few days I was sent for again. The fore and upper part of the breast had sloughed away. She was still poulticing. After she got well, there was no more gland there than in the palm of her hand. She had other children, but only one breast.

Had the cold been applied earlier, or, even too late wholly to prevent suppuration, the inflammation could have been so held in check that the gland would have been unharmed, so far as its future usefulness would have been concerned. I would not have taken time with this idea that you cannot set bounds to the suppurating process, were it not that this opinion is strongly held, and is the real reason for applying warm poultices, so as to have it over as soon as possible.

So far as I have ascertained the opinions of the physicians on this subject, I may say that they, nearly all, dread to apply cold to the abdomen or chest of a lying-in-woman. I have covered the distended belly of a wowan ill with peritonitis with snow for nearly two days, though when called to her she had been kept very warm, and had had warm poultices applied, nor did I fear the least danger for the change. Why not? Because I know full well that, whenever the human body, as in fever, is above the natural temperature, you may apply cold to every part of it which is so heated, until it is reduced to the ordinary, natural heat, without the least fear of producing chilliness; so, with local affections—inflammations I should say—the ice may be applied safely as long as the heat of the part is above the health standard. To apply cold water and ice to an inflamed breast, or to any other local inflammation, in which the part has its temperature raised, is safe, efficient, and to the patient most comfortable. Nothing that I have ever tried has given me so much satisfaction and my patient so much comfort in the disease of which I write, as the cold applications.

As it is well to have other testimony than mine, I quote the following from page 151, *Philadelphia Medical and Surgical Reporter*, of August 16th, 1879. A writer, in the *British Medical Journal*, says:

"The suggestion of treating threatened inflammation of the mammary gland by ice was one of the most valuable hints he ever got." He thus describes its use in a case: "I used a large Chapman's spine-bag, filled with ice, which encircled the lower half of the breast. It felt very cold for a minute or two, then a considerable quantity of milk was shot out as from a syringe, (no milk had flowed before), the pain abated, and in an hour was almost gone. I now renewed the ice in the bag, and the patient kept it closely applied with her arm, which was protected from the cold by a folded towel. Next morning I found her hugging the ice-bag, and loud in its praise. She continued suckling her infant, but she suggested that it should not be put to the breast oftener than two or three times in twenty-four hours. On the fourth day, after commencing with the ice, the most careful examination failed to detect anything wrong in the breast,and she is now quite well and nursing her child. No other remedies were used."

I am glad to have this testimony, for, all over the country, and I may say, in the city of Philadelphia, the old treatment by astringent washes, by poultices and plasters continues to be practised. There is no better way to apply the ice than to put it into a bladder with just enough water to float it, or just to form a kind of water cushion, that will fit the inflamed part nicely. It is not necessary to put two thicknesses of muslin between the bladder and the breast, it is not too cold without any, but a single thickness is useful to keep the bladder in place more readily. There are certain conditions of the nipple in which the iced water, in a small bladder, produces great comfort. I have never met with a single patient suffering from inflammation of the breast, and who has been treated by applications of ice and water, but has given utterance to the great relief which they brought to her. There is infinite satisfaction in bringing relief to our patients by remedies acting so promptly, so efficiently, that they cannot but recognize whence it comes.

Since the above paper was published, I have received the following letter from Dr. Helen L. Betts, an accomplished physician of several years practice—a graduate of the "Women's Medical College" of Philadelphia, in which the value of the remedy is so clearly set forth, and my testimony to its efficacy so strongly endorsed, that I have obtained the author's permission to publish it:

JAMAICA PLAIN, BOSTON, MASS., Feb. 16th, 1881.

*Dr. Hiram Corson:*

DEAR DOCTOR—Having just read your article on Mammary Abscess with exceeding interest, I wish most earnestly that I might shake hands with you over the mode of treatment. If you will allow me it will be a real pleasure to corroborate your statement by a shorter, though not less satisfactory experience. For several years I had used cold water compresses in simple congestion of the breasts incident to pregnancy and its uniform safety and efficacy so impressed me that one year ago I began its use in incipient abscess. When the treatment suggested itself to me, and good sense commended the plan, I was surprised to find how involuntarily we turn for assurance to the former experience, either of ourselves or of others, but having *neither* before me I turned—timidly at first I confess—from past practice with its poultices, plasters and prejudices, and looked squarely at physiological facts. *Heat* is one prominent symptom, and *the* important factor in the abnormal metabolic tissue changes which take place so rapidly in suppuration. *Cold* is the most natural direct and efficient antagonistic agent that can be applied in the conditions, and I applied it, and never again hesitated. Half an hour's experience will make a brave convert of the most timid practitioner, to say nothing of the suffering patient and prejudiced grandmother and nurse; all lifted together from pain and dread into ease, and the joy and confidence of success. At first I used warm flannels to other parts of the body, and gave small portions of hot sling at short intervals, and sometimes quinia, but have found that these precautions are unnecessary. The cold *alone* is *sufficient* and *safe*, though in case of a frail, anæmic woman, the stimulation is, of course, a valuable adjunct.

Some of my cases have been so typical that I believe you will be interested in them. One was a lady of forty years, with her second child of four months. She took cold by putting her hands in cold water, and in thirty-six hours about one-fourth the right breast was a *firm lump*, red, hot and tender, with angry lines streaking up to the shoulder, and rapidly growing more painful under hot applications. Iced napkins were used and the pain immediately relieved, and in thirty hours the *induration* was gone. Another was a German woman whom

I attended in her second confinement, 45 years old; she told me that with her first child there had been an excessive secretion cf milk, and the right nipple, which was retracted had become excoriated, and finally an abscess formed involving about one-third of the gland, and incisions had been made in several places and *directions.* I saw that both mammæ were enormous, and the right retracted nipple looked *ugly*, but being strong in my faith in cold as *sure safety* in worst conditions I determined to forestall danger by forearming both patient and nurse. So I charged the latter to give fluids moderately and watch the breast *closely*, and on the first appearance of unnatural heat or hardness to apply the *cold.* Taking a napkin at the time, and instructing her minutely how to proceed. This was early in the morning of the second day; unfortunately I was detained from the case for thirty-six hours, and when I visited her late in the evening of the next day, I found the poor woman in great distress from pain and apprehension. Immediately after my call, the day before the breast began to grow hot and hard, and now the whole gland stood out like a rock. The part beyond the cicatrices forming a hard angry nodulated mass, which for thirty-four hours had been growing more painful and threatening in spite of all the teas, lotions, poultices and fomentations prescribed by the neighborhood. When interrogated as to the *cold applications* the nurse said they were all *afraid to use them.* For forty-five minutes I applied ice cold napkins with my own hands, changing them every five or ten minutes as they became heated, when at the end of that time the breast was so eased, soft and cool that it was not hard to persuade both nurse and patient to continue the treatment which resulted mcst favorably.

Another case of peculiar interest is that of a woman delivered at one of our city hospitals, who two months after presented herself at the New England Hospital for Women, with an immense subglandular abscess. This was evacuated, but other collections formed and burrowed, and were incised until the breast was literally riddled and suppuration continued to such an extent that a counsel was held to consider the propriety of removing the entire breast. Dr. Samuel Cabot advised to *slash* the gland and also to make subglandular drainage by passing a seton under the whole breast. This he did, but not proving sufficient he then dilated seven of the sinuses and strapped the breast firmly down. But diptheritic deposit soon covered the openings and lined the sinuses, and the discharge became so feted and abundant in spite of disinfectant infectious, and the best tonic medication, that the woman's life was imperilled, and Dr. C. advised to dilate again, and to pass twisted bands of carbolized charpie through the main sinuses. After this operation the discharge diminished, convalescence was established, and the woman went home. A short time, however, after the patient went out she *took cold in her breast*, and when called to the unfortunate woman I found her trembling with apprehension. Some of the sinuses were still unhealed and discharging. She had been chilly for twenty-four hours. The pain in the part was excruciating, and the breast was swollen to its utmost capacity, while the old purple cicatrices that seamed the whole gland were *firing up* with a lurid tint, which in the light of past experience, was alarmingly suggestive. But I immediately swathed the angry threatening member in iced napkins, *changed often*, and the result was as *prompt* as *satisfactory*. For sore nipples and the irritation following, I have found nothing equal to cold. I might cite a large number of cases where in former pregnancies abscess had resulted from excoriation of nipples, and when called to the patients conditions were fast leading on to the same painful result, but the *cold* has *invariably* relieved, when ice bags cannot be had. I have found an excellent way to apply the cold in freezing weather is by stiffening the napkins at the window and then wrapping them smoothly about the breast.

The length of my letter startles me somewhat, but I do believe, my kind, good sir, that you will excuse the liberty assumed in writing you. Your article interested me exceedingly, and was the first I had seen on the subject.

With sincere esteem and gratitude, yours,

HELEN L. BETTS.

